# Gamma-Aminobutyric Acid and Glutamate Concentrations in the Striatum and Anterior Cingulate Cortex Not Found to Be Associated with Cognitive Flexibility

**DOI:** 10.3390/brainsci13081192

**Published:** 2023-08-11

**Authors:** Ann-Kathrin Stock, Annett Werner, Paul Kuntke, Miriam-Sophie Petasch, Wiebke Bensmann, Nicolas Zink, Anna Helin Koyun, Boris B. Quednow, Christian Beste

**Affiliations:** 1 Cognitive Neurophysiology, Department of Child and Adolescent Psychiatry, Faculty of Medicine, TU Dresden, D-01309 Dresden, Germany; miriamsophie.petasch@ukdd.de (M.-S.P.); wiebke.bensmann@ukdd.de (W.B.); nicolas.zink@ukdd.de (N.Z.); annahelin.koyun@ukdd.de (A.H.K.); christian.beste@ukdd.de (C.B.); 2 Biopsychology, Department of Psychology, School of Science, TU Dresden, D-01062 Dresden, Germany; 3 Institute of Diagnostic and Interventional Neuroradiology, TU Dresden, D-01309 Dresden, Germany; annett.werner@ukdd.de (A.W.); paul.kuntke@ukdd.de (P.K.); 4 Experimental and Clinical Pharmacopsychology, Department of Psychiatry, Psychotherapy and Psychosomatics, Psychiatric Hospital, University of Zurich, 8032 Zürich, Switzerland; boris.quednow@bli.uzh.ch; 5 Neuroscience Center Zurich, Swiss Federal Institute of Technology Zurich, University of Zurich, 8032 Zürich, Switzerland

**Keywords:** ACC, cognitive flexibility, GABA, glutamate, MRS, striatum, task switching

## Abstract

Behavioral flexibility and goal-directed behavior heavily depend on fronto-striatal networks. Within these circuits, gamma-aminobutyric acid (GABA) and glutamate play an important role in (motor) response inhibition, but it has remained largely unclear whether they are also relevant for cognitive inhibition. We hence investigated the functional role of these transmitters for cognitive inhibition during cognitive flexibility. Healthy young adults performed two paradigms assessing different aspects of cognitive flexibility. Magnetic resonance spectroscopy (MRS) was used to quantify GABA+ and total glutamate/glutamine (Glx) levels in the striatum and anterior cingulate cortex (ACC) referenced to N-acetylaspartate (NAA). We observed typical task switching and backward inhibition effects, but striatal and ACC concentrations of GABA+/NAA and Glx/NAA were not associated with cognitive flexibility in a functionally relevant manner. The assumption of null effects was underpinned by Bayesian testing. These findings suggest that behavioral and cognitive inhibition are functionally distinct faculties, that depend on (at least partly) different brain structures and neurotransmitter systems. While previous studies consistently demonstrated that motor response inhibition is modulated by ACC and striatal GABA levels, our results suggest that the functionally distinct cognitive inhibition required for successful switching is not, or at least to a much lesser degree, modulated by these factors.

## 1. Introduction

The ability to flexibly select responses is a major prerequisite for successful goal-directed behavior [1,2]. From a functional neuroanatomical perspective, fronto-striatal networks have been suggested to play an important role in cognitive flexibility, which has also been corroborated by several lines of research [3,4,5,6,7,8,9]. However, the functional role of the neurobiochemical properties of these structures is less clear, even though it is of critical relevance from a neurobiological point of view:

The basal ganglia, and the striatum in particular, contribute to response selection and cognitive control processes [10,11]. It has been stated that this largely rests upon the microstructural anatomy of the basal ganglia and the neurotransmitters that are abundant in the striatum [12]. GABAergic medium spiny neurons (MSNs) constitute the majority of striatal cells [13] and create a dense inhibitory feedback network with neighboring MSNs, thus creating a so-called “winner-take-all” network [14]. For this striatal network, computational accounts and empirical data have suggested that particularly the GABAergic modulation is central for response selection [12,15,16,17,18,19]. It is assumed that competing actions become suppressed as a consequence of this strong GABAergic modulation, so the network converges to a single winner [20]. In other words, the striatal network is able to suppress action plans and response options that are no longer needed and thereby enable a fast selection of different responses. Aside from GABA (and other potentially relevant transmitters like monoamines [21,22,23,24], which we do not detail here), the glutamatergic system also plays a major role, because glutamatergic fronto-striatal synapses modulate processes in the GABAergic striatal network [13,14,25]. Specifically, striatal interneurons suppress the GABAergic MSN network state upon activation of fronto-striatal synapses and thereby establish a new network state [14,26]. Computational and empirical work has suggested that these glutamatergic mechanisms also profoundly modulate response selection and cognitive control processes [15,19,27,28,29,30].

The mechanisms outlined above suggest that for efficient response selection processes, both the GABAergic and glutamatergic system are important to be able to flexibly shift the striatal activity focus. Given that a strong glutamatergic input facilitates efficiently shifting striatal network states, it is reasonable to assume that high striatal concentrations of glutamate correlate with high cognitive flexibility. In contrast, the direction of a correlation between striatal GABA concentrations and cognitive flexibility is less clear: On the one hand, a strong GABAergic MSN network facilitates response selection [20], which may not only speed up the selection of responses, but potentially also make shifting/switching between responses faster. On the other hand, a strong GABAergic network is more stable and requires a considerable amount of glutamatergic input to be altered. Therefore, high striatal GABA levels may also impede cognitive flexibility by increasing the glutamatergic cost of switching.

The anterior cingulate cortex (ACC) is another brain structure that is involved in response selection and flexibility, and striatal functions cannot be understood without considering closely connected neocortical areas such as the ACC [31,32,33]. GABA concentrations are not only particularly high in the striatum [34], but also abundant in cingulate areas [35]. Similarly, empirical and computational evidence [36] suggests that the GABA system in the ACC is crucial for discriminating between specific inputs, allowing for efficient response execution. In line with this, anterior cingulate GABA concentrations have been shown to modulate selection and control mechanisms [37,38]. Therefore, we examined the relevance of the GABAergic and the glutamate system for cognitive flexibility processes in both the striatum and the ACC. Given that this method has repeatedly been used to investigate the functional association between amino acid neurotransmitters and cognition [28,29,30,37,38], we used magnetic resonance spectroscopy (MRS) to measure total GABA+ (GABA+ macromolecules) and Glx (glutamate + glutamine) concentrations in the striatum and the ACC of healthy human volunteers. These GABA+ and Glx concentrations were then correlated with performance in two tasks that examined different aspects of cognitive flexibility. Given that we did not have a directed hypothesis on striatal GABA effects (and thus considered both a positive and negative association between GABA levels and performance as potentially plausible), it should be noted that correlating the ratio of both neurotransmitters with performance constitutes an exploratory analysis.

A classical experimental approach to measure cognitive flexibility, and the ability to change between different responses in particular, is the task switching paradigm [2,39]. The key finding in these paradigms is that switching between different (usually cued) task rules increases processing/response times (as compared to task rule repetition) [39]. These switch costs likely reflect reconfiguration processes, interference from the previous trial, processes related to attentional shifts, goal retrieval from working memory, and the inhibition of irrelevant task sets [2]. We hypothesized that switch costs are correlated with both striatal and ACC concentrations of Glx and GABA+. Given that switching costs may further increase when task switches have to be prompted by information held in working memory [40], which also depends on fronto-striatal structures [41,42], we hypothesized that correlations between striatal and ACC concentrations of Glx and GABA+ are particularly pronounced when working memory processes are used to trigger task switches. Overall, task switching processes and possible modulatory effects of working memory are examined in the first experiment of this study.

Another important mechanism is the inhibitory control required to suppress the no-longer-relevant task set when switching from one task to another [43,44]. It can be examined with the “backward inhibition task” [44]. In this paradigm, task rules are switched each trial and the backward inhibition (BI) effect reflects the cost of reactivating a previously inhibited task set, thus reflecting the strength of the initial inhibition. As a consequence, responses are slower and less accurate when the task rule of the n-2 trial has to be reactivated in the nth trial (as opposed to when there are three different task rules in trials n, n-1, and n-2). Specifically, the backward inhibition (BI) effect measures the effect of the task set inhibition exerted during the n-1 trial on the n-2 trial. When inhibition is strong, costs to overcome this inhibition are high. Therefore, a strong BI effect impedes task performance as it diminishes the ability to perform a previously inhibited task when it becomes relevant again [45]. The BI paradigm is the second experiment of this study. As previous findings suggest that striatal GABA+ plays an important role in response inhibition [29,30], we hypothesized that striatal and ACC concentrations of GABA+ and Glx are correlated with the BI effect. Specifically, the BI effect should be smaller when striatal and ACC concentrations of GABA+ and Glx are high.

To summarize, the current study provides a thorough investigation of the role of striatal and ACC concentrations of GABA and glutamatergic processes for different facets of cognitive flexibility.

## 2. Materials and Methods

### 2.1. Study Design

#### 2.1.1. Experimental Subjects and Ethical Approval

For this study, we recruited healthy young participants from the local university (TU Dresden) using the following inclusion and exclusion criteria: age between 18 and 32, normal or corrected-to-normal vision, no reported history of psychiatric or neurologic disease, no developmental disorders or disorders that might interfere with normal brain functioning, and no medication affecting the CNS. Participants provided written informed consent before starting the experiment and received EUR 40 as a reimbursement for their participation. The study was approved by the ethics committee of TU Dresden (under the running/project number EK420092015) and conducted in accordance with the declaration of Helsinki [46]. We recruited N = 60 participants, but as one participant failed to present for the MRS appointment, the initial sample consisted of N = 59 participants.

#### 2.1.2. Experimental Design and Procedures

Depending on personal bedtime preferences, each participant underwent MRS of the striatum and ACC within three hours after getting up in the morning (starting times varied from 08:00 to 13:00) in order to minimize potential circadian differences. The MRS data collection took about 90 min. As MRS is used to quantify the overall amount of neurotransmitters in a region of interest (and not their release), participants did not have to perform any task during MRS and were simply told to “relax and wait”. Afterward, they were taken to the EEG lab, where they filled in a sociodemographic questionnaire while the EEG cap was put on their heads. Lastly, they performed the two switching tasks reported below, as well as a Simon Nogo task [47] and a mental rotation task [48]. Please note that those other two tasks assess functionally different concepts and have therefore not yet been analyzed or published. Also, please note that we decided against analyzing the effect of neurotransmitter concentrations on the available EEG data, as we had not found any effects on the behavioral data and thus expected a heightened likelihood of false positives or functionally meaningless epiphenomena, had we done so.

### 2.2. MRS Data Acquisition and Quantification

**Figure 1:**
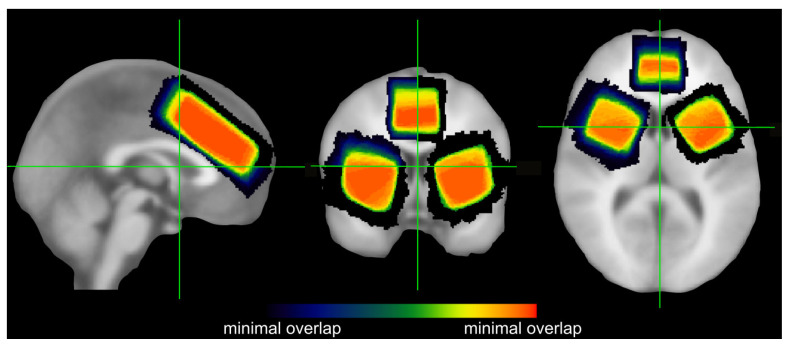
VOI placement and overlap. Illustration of the volume of interest (VOI) placements in the striatum, and the ACC of all participants whose data were included in the analyses of at least one experimental paradigm. The heat maps represent the average positioning, with warmer colors denoting more overlap between the individual positioning (i.e., zero participants overlapping is denoted by dark purple while the overlap of all included participants is denoted by reddish orange; compare color bar).

All scanning was conducted using a 3T Prisma scanner (Siemens Healthineers, Erlangen, Germany) with a 32-channel (receive only) headcoil. All MRS acquisition protocols were identical to what we used in a previous study of our group [49]. Details of the procedure are provided in the Supplementary Materials. For each of these brain regions, separate voxels of interest (VOIs) were individually positioned. The VOI used for the ACC was 20 × 30 × 60 mm in size and placed over the midline. It covered large parts of the ACC and included only small fractions outside of the ACC (see Figure 1). Additionally, two separate 30 × 30 × 30 mm VOIs were used for the left and right striatum, respectively. Unlike the ACC VOI, the striatal VOIs also included considerable fractions of adjacent structures, but given that a sufficiently large voxel of 30 × 30 × 30 mm is needed to obtain a reliable quantification of GABA+ [50], this was inevitable. When placing the striatal VOIs, we attempted to include as much as possible of the anterior and dorsal striatum (which are most important for response selection) and the putamen (which also receives motor and sensory input). As the ventral striatum is mainly characterized by limbic inputs, it was not of primary interest in the current study and not included in the striatal VOIs [30,51,52]. As we did not expect any lateralization effects (i.e., differences between the left and right striatum), the data obtained from these two VOIs were later averaged. The positioning and overlap of all three VOIs are depicted in Figure 1.

For the statistical analyses of the obtained data, we used an internal metabolite reference signal, as recommended by Mikkelsen et al. [53]. GABA+ and Glx may be referenced to either *t*Cr or NAA [50,53]. As a result of following this recommendation, the obtained measures used for statistical analyses are ratios (and hence do not have units). Both were obtained by using the corresponding “3T Siemens Edit-off Basis set” on the “edit off” spectra from the same MEGA-PRESS measurement. Yet, it is important that the reference metabolite does not have a systematic relationship with the neurotransmitter of interest and/or the other studied (in our case: behavioral) parameters [53]. In our dataset, only NAA did not significantly correlate with any of the investigated behavioral measures of both tasks (please see results section for details), so we referenced both GABA+ and Glx to NAA only. Lastly, we formed a relative measure by dividing GABA+/NAA by Glx/NAA to obtain a GABA+/Glx ratio. As GABA+ concentrations were lower than Glx concentrations in both assessed brain regions, we corrected for this by multiplying the individual GABA+ values with the factor by which the sample means of each transmitter differed in the respective region.

Different proportions of WM, GM, and CSF in the investigated VOIs could potentially also influence the metabolite levels. Therefore, we additionally quantified the fractions of these three types of tissue using the registration and segmentation functions in the Gannet toolkit (http://www.gabamrs.com/) [54]. As we found no proof of functionally relevant correlations between the fractions of GM/WM/CSF and the levels of GABA+, NAA, and GABA+/NAA in the ACC or in the striatum (please see results section for details), we refrained from controlling for these factors in our analyses.

Lastly, the MRS-derived transmitter and metabolite concentrations used for all analyses reported in the main manuscript were obtained using the recommended default of 0.15 for the DKNTMN parameter in LCModel [55]. Since this parameter allows for a quite flexible baseline curve and might thus account for a large proportion of variance in the obtained GABA+ levels/lead to an underestimation of the GABA+ values, we also explored the possibility of adjusting the DKNTMN parameter to render the baseline less flexible until this adjustment no longer improved the measuring error of GABA+. In our dataset, this was the case at a DKNTMN of 0.45. We then re-ran all MRS data analyses with the data obtained from LCModel using a DKNTMN of 0.45, essentially obtaining the same pattern of results (see Supplementary Materials). Against this background, we decided to focus on presenting and discussing the values we obtained using the default DKNTMN parameter of 0.15 in the following.

### 2.3. Experimental Tasks

This section provides an overview of the tasks used in this study. Further details are provided in the Supplementary Materials.

#### 2.3.1. Task Switching Paradigm

**Figure 2:**
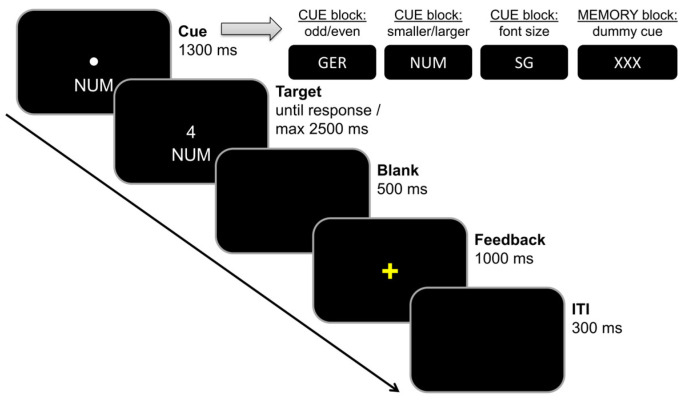
Task switching paradigm. Each trial started with the presentation of a cue in the center of the screen. In the cue block, it indicated which rule was in effect: The odd/even task (left button press for odd numbers, right button press for even numbers), the smaller/larger rule (left button press for smaller than 5, right button press for larger than five), or the font size rule (left button press for small fonts, right button press for large fonts). In the memory block, a dummy cue was shown in all trials and the order of task rules had to be updated in working memory: 3 × NUM, 3 × GER, 3 × SG. A period of 1300 ms after cue stimulus onset, the target stimulus (any number from 1 to 9, except 5) was presented above the cue stimulus until a response was given, or until 2500 ms had elapsed. A period of 500 ms after target offset, a yellow 1000 ms feedback sign was presented (“+” in case of correct responses and “−“ in case of incorrect responses). The inter-trial interval was always 300 ms long.

In order to investigate the effects of working memory load on cognitive flexibility, we used a switching task similar to that of Gajewski et al. [40], which had already been used in other studies of our group, e.g., [56,57]. The task is illustrated in Figure 2.

The experiment consisted of two distinct blocks: During the cue-based block (the first 198 trials), the presented cues informed the participants which rule was in effect. The order of cues was randomized. In 50 percent of the trials, task rules had to be switched. As a consequence, the participants had to perform a reactive form of task switching in this block, which only required minimal working memory updating (i.e., the activation of the required task rule depending on the presented cue). During the memory-based block (the last 198 trials), participants had to memorize and update a fixed order of task rules in working memory as detailed in the legend to Figure 2, while a dummy cue was presented. Due to the fixed sequence of task rules in this block, two thirds of the trials were repeat trials and one third of the trials were switch trials. As a consequence, the participants had to perform a proactive form of task switching in this block, which required self-initiated working memory updating (i.e., the activation of the required task rule without any help/based on a fixed task rule order).

Accuracy and response times were recorded for behavioral data analyses. In order to minimize the effect of extreme outliers in single trial responses, only trials with RTs between 100 ms and 2000 ms were used to determine mean hit RTs and accuracy.

#### 2.3.2. Backward Inhibition Paradigm

**Figure 3:**
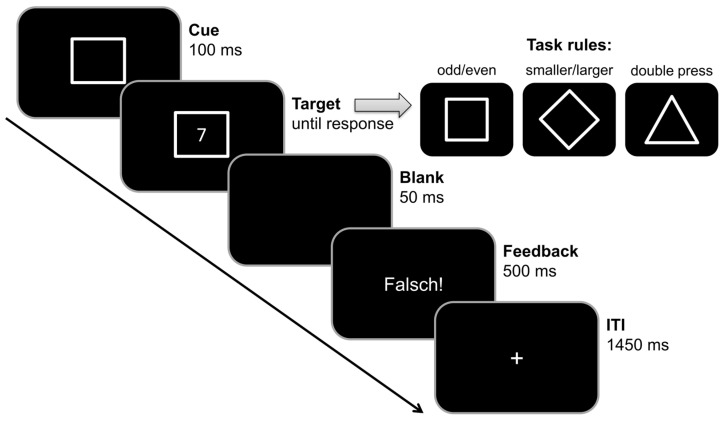
Backward inhibition paradigm. Each trial started with the presentation of a cue in the center of the screen. This cue indicated which rule was in effect: A square cue indicated the odd/even task (left button press for odd numbers, right button press for even numbers). A diamond cue indicated the smaller/larger rule (left button press for smaller than 5, right button press for larger than five). A triangle cue indicated the double press rule (simultaneous button press within the first 1000 ms after target onset). A period of 100 ms after cue stimulus onset, the target stimulus (any number from 1 to 9, except 5) was presented within the cue stimulus frame until a response was given. In double press trials (but not in case of the other two task rules), a speedup sign (“Schneller!”, translating to “Faster!”) was shown above the cue frame in case no response was given within the 1000 ms after target onset. During the inter-trial interval of 2000 ms, a 500 ms feedback sign was presented in case of incorrect responses (“Falsch!”, translating to “Wrong!”). No feedback was shown in correct trials. In the inter-trial interval (ITI) a fixation cross “+” is shown.

In order to investigate the effects of BI on cognitive flexibility, we used a BI paradigm that was originally introduced by Mayr and Keele [44], and later adapted by Koch et al. [58]. This paradigm was also previously used in other studies of our group (e.g., [59,60]). The task is illustrated in Figure 3.

Each possible combination of cues/task rules and target digits was shown equally often in each block. The order of trials within each block was randomized until it matched the following rules: There were no direct repetitions of either cue or target from trial n-1 to trial n, so two consecutive trials never contained the same cue and/or stimulus. Additionally, target stimuli could not be repeated upon the first recurrence of a given cue (e.g., if the target stimulus “2” was presented with a diamond cue in a given trial, the next trial that also presented the diamond cue could not also contain the stimulus “2”). Lastly, it was required that the 12 possible triplets formed by each trial and its two preceding trials (ABA; ADA; BAB; BDB; DAD; DBD; DBA; BDA; DAB; ADB; BAD; ABD) occurred equally often in each block (±1 triplet for two triplet conditions in each block, as each of the 8 blocks consisted of 32 trials/30 triplets and thus did not allow for each of the triplet conditions to appear exactly twice). Triplets with identical task rules in trial n and n-2 were classified as backward inhibition (BI) triplets. Triplets with non-identical task rules in trial n and n-2 were classified as baseline (BASE) triplets. We only included ABA/BAB as BI triplets and DBA/DAB as BASE triplets in our analyses, as previous research had indicated that those four triplets are most suitable in order to observe the BI effect [58,59].

The accuracy of all three trials of a triplet as well as the response times of the last trial of each included triplet were recorded for behavioral data analyses. In order to minimize the effect of extreme outliers in single trials responses, only triplets with RTs between 100 ms and 2500 ms in the last trial were used to determine mean hit RTs and accuracy. Lastly, it should also be noted that due to only rating triplets with three consecutive responses as correct, the chance level of correct responses was at 12.5% (not 50%) for this paradigm.

### 2.4. Statistics

In order to ensure that both paradigms yielded the expected task effects, we conducted separate repeated-measures ANOVAs for both accuracy and hit RT measures in both tasks. For the task switching paradigm, we used block (cue vs. memory) and condition (switch vs. repeat) as within-subject factors. Additionally, we calculated the switching effect (switch minus repeat) for both blocks separately and used these measures for post hoc tests as well as correlation analyses (see below). For the BI paradigm, we used condition (BI vs. BASE) as the within-subject factor. Additionally, we calculated the BI effect (BI minus BASE) and used this measure for correlation analyses (see below). Greenhouse–Geisser corrections were applied whenever necessary. Post hoc tests did not undergo Bonferroni correction. Descriptive data are given as the mean and standard error of the mean (SEM).

To ensure that any correlations observed between the assessed transmitters and behavioral levels were indeed due to variations in the investigated transmitter (and not the reference metabolite), we started the MRS analyses by correlating absolute tCr and NAA concentrations (i.U.) and behavioral measures. To assess whether MRS-assessed transmitter levels correlated with the performance in the investigated tasks, we then performed linear correlation analyses as well as multiple linear regression analyses with the NAA-referenced MRS values as independent measures and each single behavioral measure as separate dependent variables.

Because non-significant results obtained in regular parametric testing (including linear correlation analyses) do not allow for reliable statements on whether or not the null hypothesis is more likely to be true than the alternative hypothesis, we additionally conducted Bayesian linear correlation analyses for non-significant results.

## 3. Results

### 3.1. Exclusion of Participants and Outlier Values

After collecting the data, we inspected the MRS data and excluded n = 4 participants from subsequent data analyses due to the lack of usable MRS data and deleted extreme outlier values/bad MRS data in n = 11 participants (but kept the other/non-outlier values of those n = 11 participants and still included them in the analyses reported below). We also inspected the behavioral performance data and excluded n = 2 participants from the task switching paradigm analyses due to extreme (low) outliers in accuracy rates and excluded n = 4 participants from the backward inhibition paradigm analyses due to the lack of participation, outlier (high) reaction times, or outlier (low) accuracy. Further details are provided in the Supplementary Materials.

#### 3.1.1. Task Switching Paradigm: Sample Characterization

On average, the analyzed n = 54 participants (30 of them female) were 24.7 ± 0.5 years old (range 18–32).

#### 3.1.2. Task Switching Paradigm: Behavioral Data

The ANOVA for accuracy revealed a significant effect of condition (F(1,53) = 4.961, *p* = 0.030, η^2^_p_ = 0.086), η^2^_p_ with more correct responses in repeat trials (95.74% ± 0.39) than in switch trials (95.09% ± 0.51). There was no significant main effect of block (F(1,53) = 3.983, *p* = 0.051, η^2^_p_ = 0.070), or interaction between block and condition (F(1,53) = 1.578, *p* = 0.215, η^2^_p_ = 0.029).

The ANOVA for RTs revealed a main effect of block (F(1,53) = 5.660, *p* = 0.021, η^2^_p_ = 0.096), with lower RTs in the cue block (696 ms ± 18) than in the memory block (725 ms ± 20). There was also a main effect of condition (F(1,53) = 76.750, *p* < 0.001, η^2^_p_ = 0.592), with faster RTs in repeat trials (679 ms ± 17) than in switch trials (742 ms ± 20). Moreover, there was a significant interaction between block and condition (F(1,53) = 24.533, *p* < 0.001, η^2^_p_ = 0.316). Post hoc dependent-sample t-tests revealed that for switch trials, RTs were higher in the memory condition (771 ms ± 23) than in the cue condition (713 ms ± 20) (t(53) = 3.557, *p* < 0.001). There was no such significant block difference for repeat trials (t(53) = 0.151, *p* = 0.880). Further post hoc paired t-tests showed that while the switching effect (i.e., the condition effect) was significant in both blocks (all *p* < 0.001), the condition difference (switch minus repeat) was significantly greater in the memory block (91 ms ± 11) than in the cue block (35 ms ± 7) (t(53) = 4.953, *p* < 0.001).

Exploratory add-on analyses of the behavioral data with sex as an additional between-subjects factor did not reveal any significant effects of this factor (for details, please refer to the Supplementary Materials).

#### 3.1.3. Task Switching Paradigm: MRS Measures

To ensure that any (potential) correlations observed between the assessed transmitters and behavioral levels was indeed due to variations in the transmitter of question (and not the reference metabolite), we started the MRS analyses by correlating absolute tCr and NAA concentrations derived from the MEGA-PRESS “edit off” spectrum with the behavioral measures. This revealed significant correlations of the absolute tCr values with some of the behavioral measures in the task switching paradigm: The ACC absolute tCr significantly correlated with the RT switching effect in the memory condition (i.e., memory/switch minus memory/repetition; r = 0.304, *p* = 0.029) and add-on Bayesian analyses provided anecdotal evidence in favor of the alternative hypothesis (BF01 = 0.852). All other correlations between absolute tCr and behavioral values were non-significant (all *p* ≥ 0.051; all BF01 ≥ 1.405). Given this weak evidence, we consider the observed correlation to most likely be incidental/a false positive. In order to avoid accidentally biasing the analyses, it is however important to not reference the transmitters of interest to tCr in this case. In contrast to this, NAA reference values did not significantly correlate with any behavioral measure in the task switching paradigm (all *p* ≥ 0.057) and all Bayesian analyses for the NAA correlations were more in favor of the null hypothesis than of the alternative hypothesis (all BF01 ≥ 1.511). Based on this, we decided to reference Glx and GABA+ to NAA for the following analyses.

We furthermore checked whether differences in the fractions of grey matter (GM), white matter (WM), and cerebrospinal fluid (CSF) in the voxels correlated with the NAA-referenced neurotransmitter levels or the GABA+/Glx ratio. Doing so, we found no significant correlations (all *p* ≥ 0.105). For all of these non-significant correlations, there was more evidence for the null hypothesis than for the alternative hypothesis (all BF01 ≥ 2.375). Given these results, it was not necessary to correct for differences in VOI composition.

**Figure 4:**
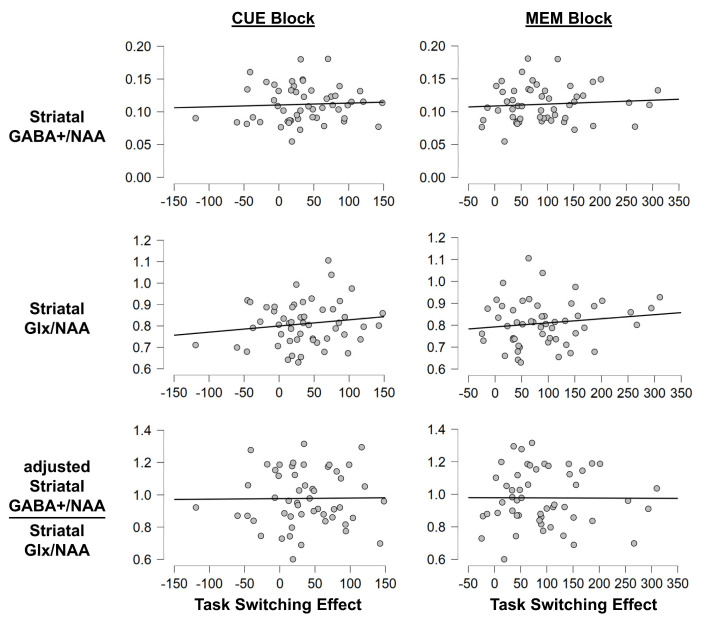
Correlation plots for the task switching paradigm (striatum). Exemplary correlation plots illustrating the lack of significant correlations between striatal neurotransmitter levels and behavior. The x-axis denotes the switch costs on hit RTs in ms (i.e., SWITCH minus REPEAT). The Y-axis denotes the neurotransmitter levels.

**Figure 5:**
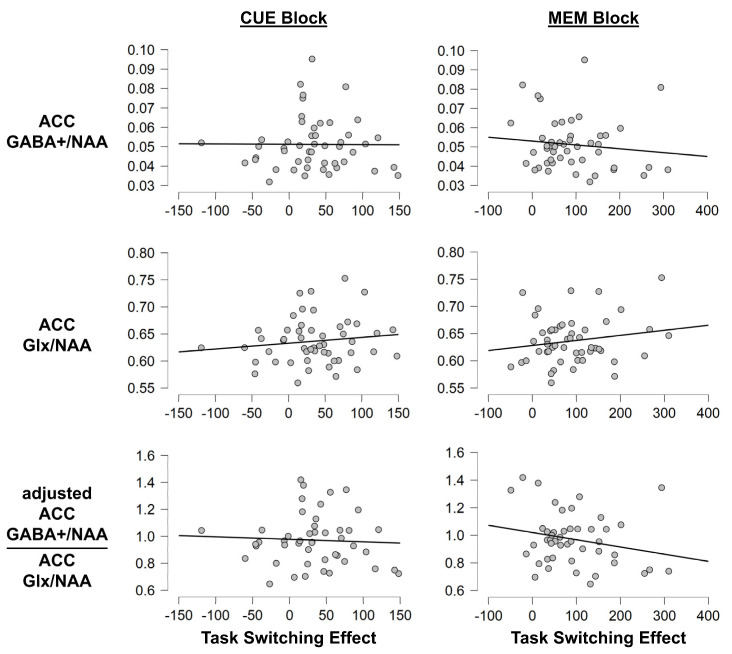
Correlation plots for the task switching paradigm (anterior cingulate cortex, ACC). Exemplary correlation plots illustrating the lack of significant correlations between anterior cingulate neurotransmitter levels and behavior. The x-axis denotes the switch costs on hit RTs in ms (i.e., SWITCH minus REPEAT). The y-axis denotes the neurotransmitter levels.

To investigate whether MRS-assessed transmitter levels correlated with performance in the task switching paradigm, we ran linear correlation analyses. They revealed a significant correlation between GABA+/Glx in the ACC and hit RTs of switch trials in the memory block (r = −0.310; *p* = 0.034). Still, an add-on Bayesian analysis only provided small anecdotal evidence for the alternative hypothesis (BF01 = 0.937). Aside from this effect, there were no other significant correlations between any of the assessed behavioral parameters and GABA+/NAA or Glx/NAA or GABA+/Glx in either the ACC or striatum (all *p* ≥ 0.127). Of note, the add-on Bayesian analyses for all of the other correlations were also more in favor of the null hypothesis than the alternative hypothesis, even though evidence was sometimes only on an anecdotal level (all BF01 ≥ 2.763). To illustrate this, Figure 4 and Figure 5 depict the correlation between switch costs and the assessed neurotransmitters.

We further performed multiple linear regression analyses with all MRS values as independent and each single behavioral measure as separate dependent variables to further confirm our results of the correlation analyses.

We neither found the GABA+/NAA MRS measures (all *p* ≥ 0.262, adj. R2 ≤ 0.016), nor the Glx/NAA MRS measures (all *p* ≥ 0.137, adj. R2 ≤ 0.042), nor the GABA+/Glx ratio measures (all *p* ≥ 0.140, adj. R2 ≤ 0.045) to be significant predictors for any behavioral measure. Furthermore, additional Bayesian regression analyses indicated that there is at least strong evidence for the null hypothesis compared to the alternative hypothesis for the GABA+/NAA MRS measures (all BF01 ≥ 13.583), and at least substantial evidence for the Glx/NAA MRS measures (all BF01 ≥ 7.426) and for the GABA+/Glx ratio measures (all BF01 ≥ 7.011).

#### 3.1.4. Mayr Switch Paradigm: Sample Characterization

The n = 51 participants (27 female) included in the analyses were 24.7 ± 0.5 years old (range 18–31).

#### 3.1.5. Mayr Switch Paradigm: Behavioral Data

The ANOVA for accuracy revealed no significant effect of condition (F(1,50) = 2.575, *p* = 0.115, η^2^_p_ = 0.049). However, the ANOVA for RTs revealed a significant main effect of condition (F(1,50) = 44.819, *p* < 0.001, η^2^_p_ = 0.473). As RTs were longer in the backward inhibition (BI) condition (773 ms ± 19) than in the baseline (BASE) condition (735 ms ± 18), this condition difference provides evidence of the typical BI effect. Based on this finding, we decided to only use RTs for the subsequent correlation analyses relating task performance to the obtained MRS measures.

Exploratory add-on analyses of the behavioral data with sex as an additional between-subjects factor did not reveal any significant effects of this factor (for details, please refer to the Supplementary Materials).

#### 3.1.6. Mayr Switch Paradigm: MRS Measures

To ensure that any (potential) correlations observed between the assessed transmitters and behavioral levels was indeed due to variations in the transmitter of question (and not the reference metabolite), we started the MRS analyses by correlating absolute tCr and NAA concentrations and behavioral measures. Those correlation analyses revealed only non-significant results (all *p* ≥ 0.088) and all Bayesian analyses were more in favor of the null hypothesis than of the alternative hypothesis (all BF01 ≥ 2.151), thus suggesting that there was no meaningful correlation between either tCR or NAA and performance. Against this background, we decided to use NAA-referenced values for further analyses in the BI paradigm as well, as this yields better comparability with the task switching paradigm.

**Figure 6:**
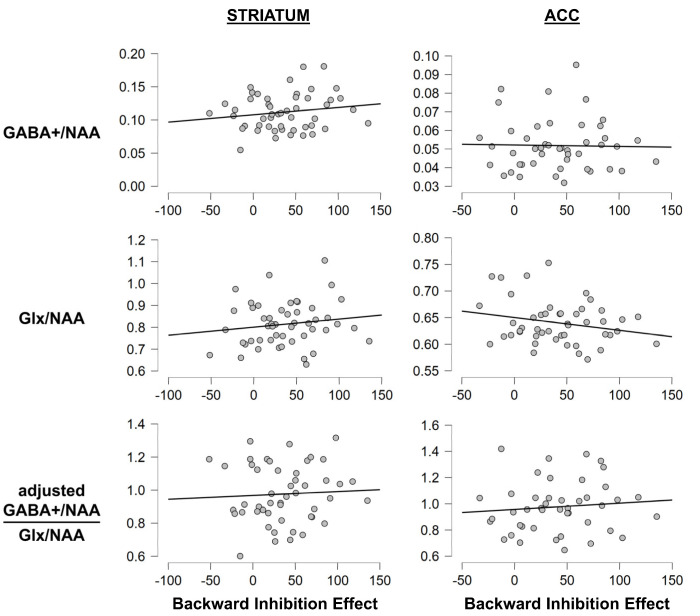
Correlation plots for the backward inhibition paradigm. Exemplary correlation plots illustrating the correlations between neurotransmitter levels and behavior. The x-axis denotes the BI effect on hit RTs in ms (i.e., BI minus BASE). The y-axis denotes the neurotransmitter levels. The transmitter levels in the striatum are illustrated in the left column and those in the ACC are illustrated in the right column. The grey asterisk in the middle right graph denotes the only obtained significance. It should however be noted that this significance would not survive corrections for multiple testing and was not substantiated by the other analyses.

We also correlated GABA+/NAA, Glx/NAA, and the GABA+/Glx ratio with behavioral RT measures. Doing so found no significant correlations between any of the assessed behavioral parameters and GABA+/NAA or Glx/NAA or GABA+/Glx in either the ACC or striatum (all *p* ≥ 0.118), and add-on Bayesian analyses provided evidence for the null hypothesis (all BF01 ≥ 2.630), even though it was only anecdotal in one case. To illustrate this, Figure 6 depicts the correlation between the BI effect and the assessed neurotransmitters.

We further performed multiple linear regression analyses with the MRS values as independent and each single behavioral measure as separate dependent variables to further confirm our results of the correlation analyses. We neither found the GABA+/NAA MRS measures (all *p* ≥ 0.433, adj. R2 ≤ 0.007), nor the Glx/NAA MRS measures (all *p* ≥ 0.297, adj. R2 ≤ −0.011), nor the GABA+/Glx ratio measures (all *p* ≥ 0.681, adj. R2 ≤ −0.030) to be significant predictors of any behavioral measure. Further Bayesian regression analyses were also more in favor of the null hypothesis than of the alternative hypothesis and indicated that there was at least strong evidence for the null hypothesis for the GABA+/NAA MRS measures (all BF01 ≥ 20.977) and the Glx/NAA MRS measures (all BF01 ≥ 15.040), and at least very strong evidence for the GABA+/Glx ratio measures (all BF01 ≥ 31.387).

### 3.2. Summary of Main Results

Taken together, we found typical task effects (i.e., evidence for the switching effect and the BI effect), but the data did not confirm our hypotheses that GABA+ levels, Glx levels, or their ratio in the ACC or striatum correlate with task switching performance, or with BI: correlation analyses did not reveal significant correlations of either transmitter, or their ratio, with any of the relevant behavioral measures, except for a single correlation between GABA+/Glx in the ACC and response times in switch trials in the memory block of the switching task. Yet, it needs to be noticed that this result was obtained without correcting for multiple testing and Bayesian analyses failed to provide convincing evidence for the alternative hypothesis being true. As GABA+ and Glx levels as well as their ratio also did not predict any of the behavioral measures in the subsequent multiple linear regression analyses, we deem it safe to state that we found no functionally relevant effects.

## 4. Discussion

The goal of the current study was to examine the role of the striatal and ACC concentrations of GABA and glutamate for different facets of cognitive flexibility. To this end, we performed MRS to examine structure-specific GABA+ and Glx levels, as well as their ratio. Behavioral data obtained from two cognitive flexibility tasks (a cue- vs. memory-based task switching paradigm as well as a BI paradigm) were correlated with the MRS data. For both paradigms, the behavioral data replicated well-known task-related effects, that is, higher switch costs for memory-based than for cue-based task switching (paradigm 1) and a clear BI effect (paradigm 2). We also found that the memory block of the task switching paradigm, which requires proactive switching, resulted in slower but more accurate responses than the cue block, which requires reactive switching. However, almost all of the examined behavioral parameters, including measures for the switching effect and the BI effect, were not correlated with striatal or ACC concentrations of GABA+ and Glx, or their ratio. Add-on Bayesian analysis further substantiated that the evidence for the null hypothesis (i.e., no correlation between GABA+, or Glx, or their ratio with behavioral performance) was greater than that for the alternative hypothesis in all but one case. Complementing this picture, the only correlation that turned out significant would not have survived corrections for multiple testing, could not be substantiated in add-on Bayesian testing, and was not confirmed by subsequent regression analyses. Taken together, these data suggest that in healthy young adults, GABA+ and Glx levels do not seem to affect task-switching performance and BI—at least not in a significant linear manner.

We focused our examination on the striatum as theoretical considerations and computational accounts had previously demonstrated striatal GABAergic and glutamatergic modulation to be central for response selection [12,12,14,15,16,17,18,19,26]. Similarly, the ACC’s GABA system had previously been shown to be crucial for discriminating between specific inputs, thereby allowing for efficient response execution [36]. Switch costs can emerge as a consequence of different processes [2]. Some findings suggest that processes of response execution or processes in the cascade of motor response preparation and execution might have a major impact on switch costs [61,62,63,64,65,66,67]. Still, there is an intense debate about the relative importance of “cognitive” and “motor” aspects for the emergence of switch costs [66]. If motor aspects were (most) relevant, neurotransmitter variations in functionally relevant neuroanatomical structures, that are well known to modulate motor response selection processes [28,29,38], should have been associated with switch costs. Given that this was clearly not the case for the striatum and the data obtained for the anterior cingulate cortex also did not sufficiently support this assumption, it may be concluded that motor re-programming processes are less important for switch costs than previously thought.

Based on our findings from the first paradigm (cue- vs. memory-based task switching), it can of course not be ruled out that the concentration of the GABAergic and glutamatergic system in other brain structures, that were not examined in the current study, may still be relevant. Nevertheless, this is unlikely to be the case for prefrontal areas as fronto-striatal networks have a funnel-like architecture with all prefrontal regions projecting to the basal ganglia [31,32]. In this context, the striatum represents a hub where information from the neocortex converges. The lack of association or modulatory effects suggest that GABA+ and Glx levels in fronto-striatal networks are irrelevant for processes of cognitive flexibility. Of note, previous studies that used a comparable MRS approach and reported an association between striatal GABA+ concentrations and cognitive control functions found these associations in experimental paradigms with a clear motor control component; i.e., a Go/Nogo response inhibition task [29,38] and a Simon task [28]. Therefore, the most likely theoretical implication of the current findings is that motor-related processes, which are known to depend on GABA levels in the investigated brain regions, seem to be less important for switch costs than other processes, which are not directly linked to motor response inhibition.

Corroborating this interpretation, the data from the BI experiment suggest that (non-motor) “inhibitory control” processes, although relevant for cognitive flexibility [2,44], are also not associated with striatal or ACC concentrations of GABA+, or with striatal concentrations of Glx. And while we found a single correlation between ACC concentrations of glutamate and the BI effect, this finding was nothing more than anecdotal and cannot in good conscience be claimed to provide any reliable evidence for a functional link. At first glance, this lack of effects may seem at odds with previous MRS studies showing the relevance of the striatal and ACC GABA systems for response inhibition [29,38]. Yet, “inhibitory control” is not a single unitary function. It comprises both “behavioral inhibition” and “cognitive inhibition”, which can be functionally dissociated [68]. Behavioral inhibition refers to (i) Response inhibition, which can further be subdivided into postponing, withholding, and cancelling a given action. Moreover, (ii) reversal learning and (iii) delayed gratification also fall under the term of behavioral inhibition [68]. In contrast to this, cognitive inhibition refers to the inhibition of unwanted memories, thoughts, perceptions, and emotions [68]. In the BI task, the “inhibition” component of the task refers to the suppression of task sets, i.e., the effect of the n-2 task set inhibition exerted during the n-1 trial on the n-2 trial [44,45]. It is hence possible that striatal and ACC concentrations of GABA and glutamate are only relevant for “behavioral inhibition” (as assessed in motor tasks like the NoGo paradigm), but do not play a major role in ”cognitive inhibition” (as assessed in cognitive flexibility tasks).

Lastly, it should be noted that the lack of findings in our study mainly refutes the hypothesis that overall concentrations of GABA+ and Glx in the striatum or ACC determine switch costs to a relevant degree. As the method of MRS neither allows for distinguishing between free and vesicular GABA, nor allows for assessing differences in active signaling during task performance (e.g., inter-individual differences in tonic vs. phasic firing patterns, the amount of transmitter release or reuptake, or postsynaptic receptor density), it is still conceivable that differences in any of these factors might be associated with switch costs in the absence of differences in absolute transmitter concentrations. Likewise, it is well possible that other neurotransmitter systems, including different monoamines and especially the dopamine and serotonin system [24,69,70], might play a much more important role than the assessed amino acid transmitters. For example, prefrontal serotonin levels are known to modulate prefrontal input into the striatum [21,24], while dopamine is known to modulate GABAergic signaling in both the striatum [71,72] and the ACC [73,74]. While further research on this nexus is still needed, it has become apparent that dopamine plays a key role in inhibitory aspects of behavioral control and for set shifting, while serotonin appears to be indispensable for cognitive flexibility and noradrenaline is key to attention shifting [24]. While such dopaminergic modulation has been demonstrated to alter the release of GABA and/or the sensitivity of postsynaptic neurons to these transmitters [74,75], it is unlikely to up- or down-regulate the overall levels of GABA or glutamate in these brain areas, and should therefore not be reflected by changes in the assessed MRS-measures, as well. While it is unfortunately impossible to quantify dopamine levels using MRS, it would be interesting to try to investigate the combined effects of functional differences in the storage and release of amino acid and monoaminergic neurotransmitters on both motor and cognitive inhibition in future studies.

Lastly, it is important to discuss evident limitations. As explained in the Methods section, the striatal VOI also included considerable fractions of adjacent structures, but given that a sufficiently large voxel of 30 × 30 × 30 mm is needed to obtain a reliable quantification of GABA+ [50], this was inevitable. Furthermore, it needs to be noted that MRS quantifies the total GABA or glutamate levels together with macromolecules (GABA+) and glutamine (Glx), which may also vary and thus increase the overall variance. Also, MRS cannot selectively identify functional extracellular or synaptic concentrations. This means that changes in transmitter release during task performance cannot be assessed. So, even though the MRS cannot be assessed “online” (i.e., during task performance), due to the duration of the measurement, it should still provide an insight into the current state during task performance, as the MRS was assessed in close temporal proximity. It is hence possible that the quantification of synaptic concentrations or receptor densities with different methods (e.g., PET) might have yielded different results. Concerning the task design, it would likely be advisable to control for the number of repeat vs. switch trials in the cue vs. memory block in the task switching paradigm in order to be able to exclude potential differences as factors contributing to the observed block differences. Given that the ratio of GABA+ to glutamate (as well as fronto-striatal connectivity) is known to change with age, especially from childhood and adolescence to adulthood [76], it would furthermore be interesting to investigate whether our findings also hold true in healthy underaged samples. Furthermore, it needs to be noted that the vast majority of our participants were students/had higher education. Given that task switching abilities have been identified as a predictor of academic success (with better task switching being associated with better academic performance later in life) [77], our sample might have been characterized by above-average task switching performance and, as a consequence, have been less varied than in a more representative population sample.

## 5. Conclusions

In summary, the study shows that striatal/ACC concentrations of GABA+ and Glx do not seem to modulate cognitive flexibility as examined by two different experimental paradigms, assessing cue- vs. working-memory-based task switching and BI. These findings have major implications for cognitive theory and neurobiology, because they suggest that behavioral and cognitive inhibition are not only functionally distinct faculties, but also depend on (at least partly) different brain structures and neurotransmitter systems. While motor response inhibition has been previously demonstrated to be modulated by ACC and striatal GABA levels, our findings suggest that the cognitive inhibition required for successful switching is not, or at least to a much lesser degree. Further combined fMRI, MRS, and PET studies will be needed to investigate the functional and neurobiochemical differences between response inhibition and cognitive inhibition in order to better understand the dissociation between those behaviorally/cognitively related, yet neurobiologically different, functions.

## Data Availability

The datasets used and/or analyzed during the current study are available from the corresponding author on reasonable request.

## Supplementary Materials

The following supporting information can be downloaded at: https://www.mdpi.com/article/10.3390/brainsci13081192/s1, Figure S1: Overlay of the spectra of all included participants for all three VOIs. Upper graph: ACC VOI. Middle graph: left striatum VOI. Lower graph: right striatum VOI. The grey bar in each graph. Figure S2: ACC model fit. Representative LCModel fit of MEGA-PRESS for the ACC in a single exemplary participant. Upper graph black curve: Residual curve (depicting the difference between the fitted and the measured curves). Lower graph red curve: Fitted curve. Lower graph black curve: Measured curve. Lower graph grey curve: Baseline curve. Right panel: Positioning of the voxel of interest in the exemplary participant. Figure S3: Striatal model fit. Representative LCModel fit of MEGA-PRESS for the striatum in a single exemplary participant. Upper graph black curve: Residual curve (depicting the difference between the fitted and the measured curves). Lower graph red curve: Fitted curve. Lower graph black curve: Measured curve. Lower graph grey curve: Baseline curve. Right panel: Positioning of the voxel of interest in the exemplary participant highlights the GABA peak(s). References [78,79,80,81] were cited in the Supplementary Materials.
