# Supplementary material for: Gamma-Aminobutyric Acid and Glutamate Concentrations in the Striatum and Anterior Cingulate Cortex Not Found to Be Associated with Cognitive Flexibility
Source: Brain Sci. 2023 Aug 11;13(8):1192. doi: 10.3390/brainsci13081192 (PMC10452168; doi:10.3390/brainsci13081192)
Supplement: Supplementary file 1 [file brainsci-13-01192-s001.zip › brainsci-2531232-supplementary.pdf]

brain sciences

MDPI

Supplementary Materials

Gamma-Aminobutyric Acid and Glutamate Concentrations in the Striatum and Anterior Cingulate Cortex Not Found to Be Associated with Cognitive Flexibility

Ann-Kathrin Stock [1,2,*,†], AnnettWerner [3,†], Paul Kuntke [3], Miriam-Sophie Petasch [1] and Wiebke Bensmann [1], Nicolas Zink [1], Anna Helin Koyun [1], Boris B. Quednow [4,5] and Christian Beste [1]

1 Cognitive Neurophysiology, Department of Child and Adolescent Psychiatry, Faculty of Medicine, TU Dresden, D-01309 Dresden, Germany; miriamsophie.petasch@ukdd.de (M.-S.P.); wiebke.bensmann@ukdd.de (W.B.); nicolas.zink@ukdd.de (N.Z.); annahelin.koyun@ukdd.de (A.H.K.); christian.beste@ukdd.de (C.B.)
2 Biopsychology, Department of Psychology, School of Science, TU Dresden, D-01062 Dresden, Germany
3 Institute of Diagnostic and Interventional Neuroradiology, TU Dresden, D-01309 Dresden, Germany; annett.werner@ukdd.de (A.W.); paul.kuntke@ukdd.de (P.K.)
4 Experimental and Clinical Pharmacopsychology, Department of Psychiatry, Psychotherapy and Psychosomatics, Psychiatric Hospital, University of Zurich, 8032 Zürich, Switzerland; boris.quednow@bli.uzh.ch
5 Neuroscience Center Zurich, Swiss Federal Institute of Technology Zurich, University of Zurich, 8032 Zürich, Switzerland
* Correspondence: ann-kathrin.stock@ukdd.de
† These authors contributed equally to this work.

1. Materials and Methods

1.1. Additional Details on the MRS Method

First, structural images were obtained using a high-resolution 3D T1- weighted sagittal Magnetization Prepared Rapid Gradient Echo (MPRAGE) sequence (1 mm isovoxel). These were then reconstructed for exact voxel placements. 1H-MR-spectroscopy was used to quantify brain metabolite (GABA+, Glx, NAA, tCr) concentrations in the striatum and the ACC. For each of these brain regions, separate voxels of interest (VOIs) were individ-ually positioned [Details on how the VOIs were positioned are provided in the main man-uscript]. Once the VOIs were placed, manual shimming was performed (in additional to the inbuilt shim routine) for each of the VOIs in order to further optimize spectral resolu-tion. The criterion used for this was a full width at half maximum (FWHM) value below 20 Hz for the unsuppressed water signal. To obtain GABA+ and Glx values, we then ran the CMRR (Center for Magnetic Resonance Research) MEGA-PRESS (Mescher-Garwood point-resolved spectroscopy) sequence (echo time TE/ repetition time TR = 68/2000 ms, edit ON acquisitions = 128, edit OFF acquisitions = 128) developed by Edward J. Auerbach and Małgorzata Marjańska and provided by the University of Minnesota [78,79], based on a C2P license agreement with Siemens Healthineers AG Germany.

To quantify GABA+, Glx, NAA, and *t*Cr in the obtained spectra, we used LCModel software (v6.3-1H) [80]. Basis sets for MEGA-PRESS were delivered by Ulrike Dydak`s La at Purdue University (http://purcell.healthsciences.purdue.edu/mrslab/ba-sis_sets.html). Only spectra of final adequate shim quality (FWHM of 3-7 Hz of the NAA peak) were used for the subsequent quantification in order to ensure sufficient data quality. In the entire sample, we further assessed the absolute GABA+ error estimate, as this measure typically has a higher error than Glx or the reference metabolite. Doing so, we obtained values below the 20% Cramér-Rao lower bound (CRLB or %SD) criterion [81] for the striatum (right side: 11.17 ± 1.74, left side: 10.27 ± 1.69) and the ACC (12.29 ± 2.42). An overlay of the spectra of all included participants for all three VOIs is provided in Figure S1. A representative LCModel fit of MEGA-PRESS is depicted in Figures S2 and S3.

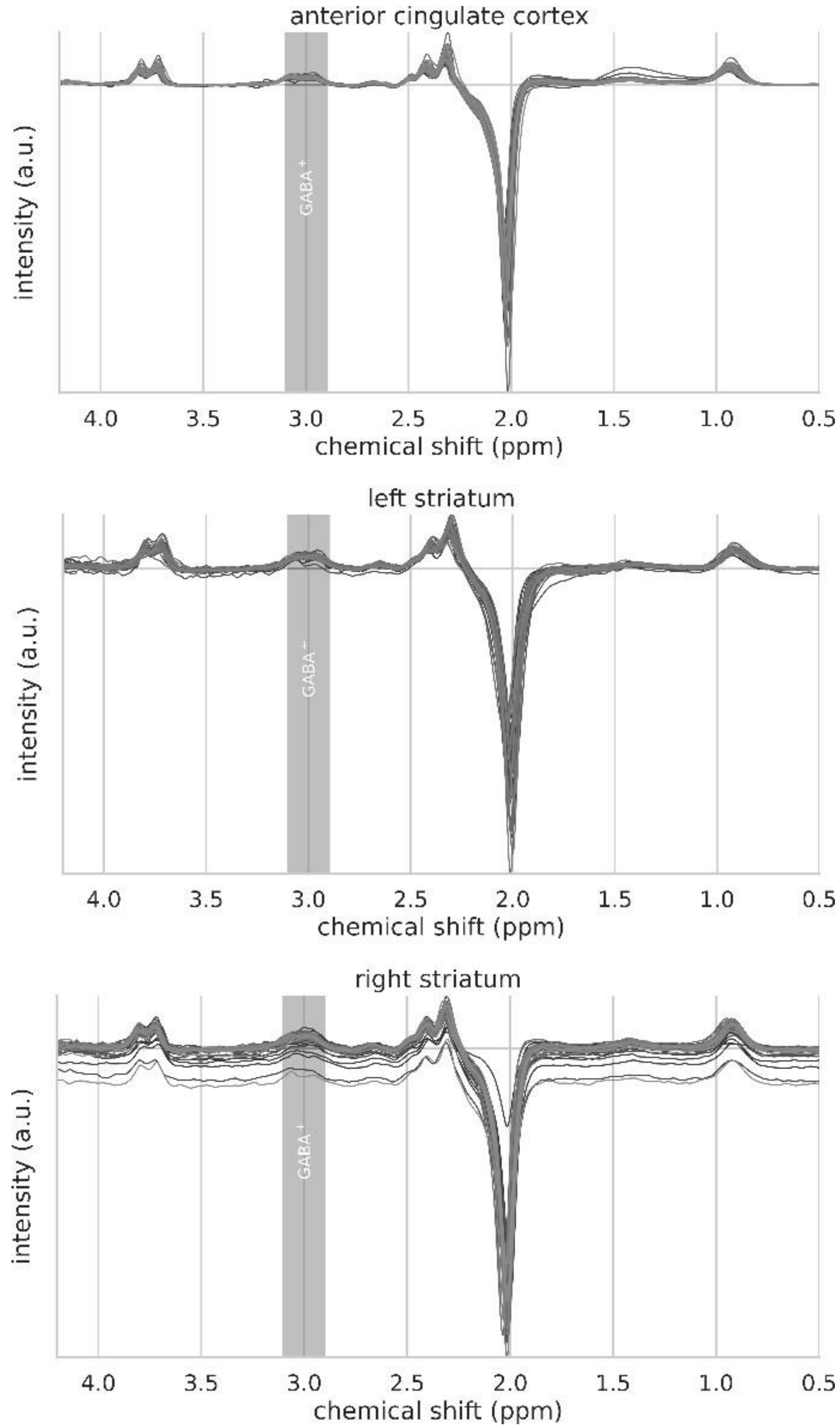

Figure S1. Overlay of the spectra of all included participants for all three VOIs. Upper graph: ACC VOI. Middle graph: left striatum VOI. Lower graph: right striatum VOI. The grey bar in each graph highlights the GABA peak(s).

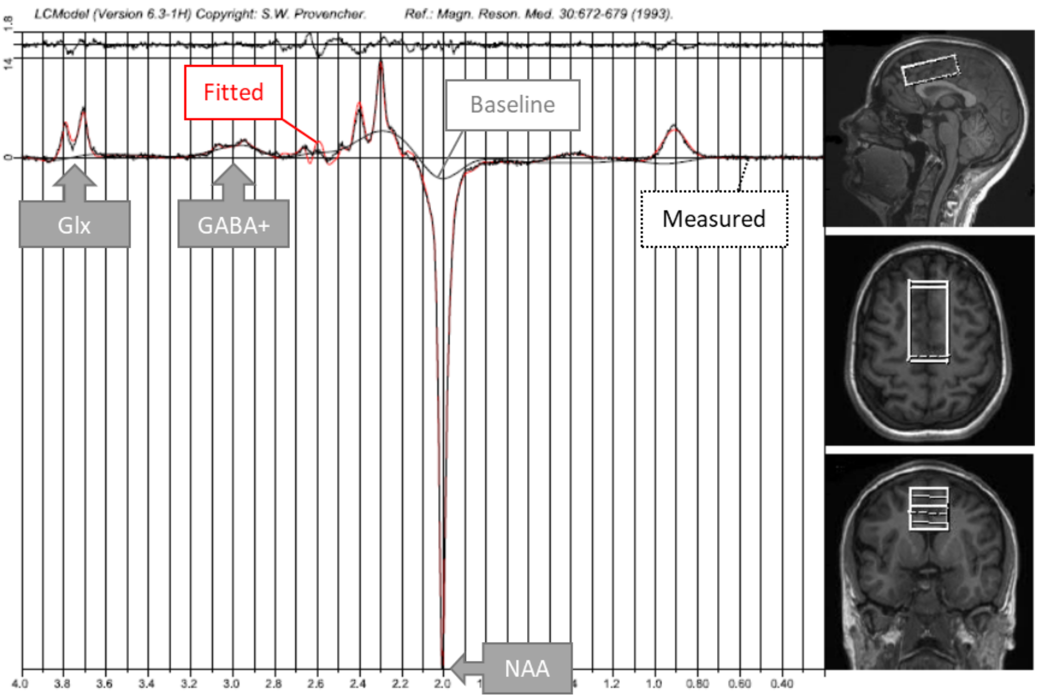

Figure S2. ACC model fit. Representative LCModel fit of MEGA-PRESS for the ACC in a single exemplary participant. Upper graph black curve: Residual curve (depicting the difference between the fitted and the measured curves). Lower graph red curve: Fitted curve. Lower graph black curve: Measured curve. Lower graph grey curve: Baseline curve. Right panel: Positioning of the voxel of interest in the exemplary participant.

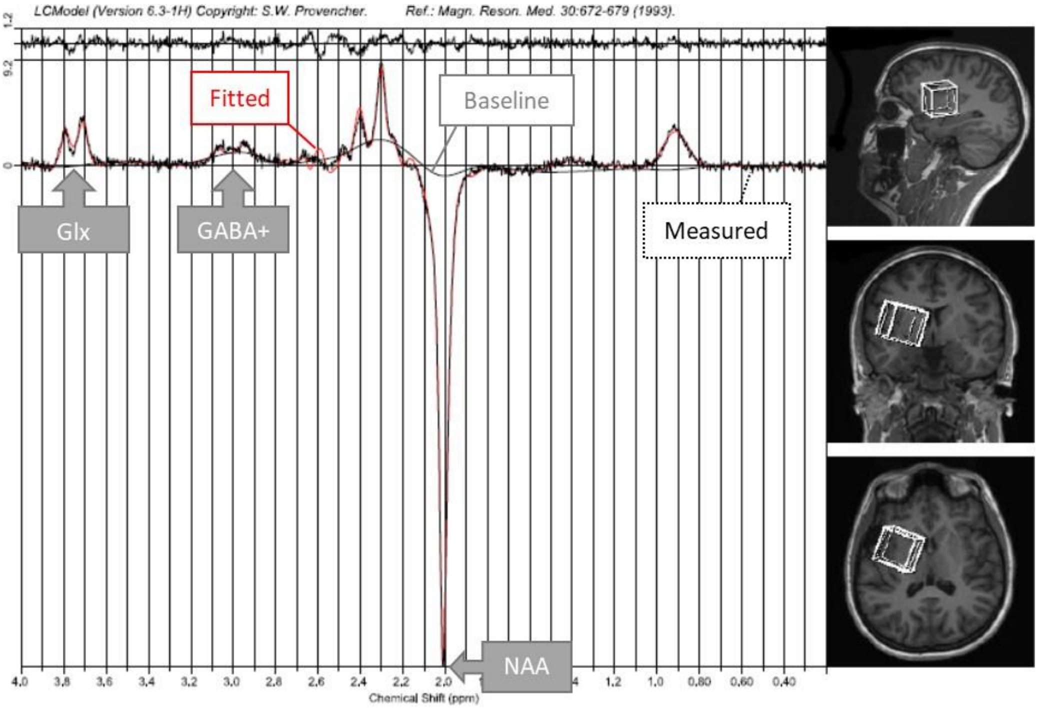

Figure S3. Striatal model fit. Representative LCModel fit of MEGA-PRESS for the striatum in a single exemplary participant. Upper graph black curve: Residual curve (depicting the difference between the fitted and the measured curves). Lower graph red curve: Fitted curve. Lower graph black curve: Measured curve. Lower graph grey curve: Baseline curve. Right panel: Positioning of the voxel of interest in the exemplary participant.

1.2. Additional Details on the Applied Experimental Tasks

The tasks were presented on a 24 inch TFT computer monitor at a viewing distance of 57 cm. The software "Presentation" (Neurobehavioral Systems, Inc.) was used for stimulus presentation. Participants responded on a regular USB keyboard with the left and right Ctrl buttons by using their left and right index fingers, respectively. Participants were seated in a separate room from the experimenters to prevent distraction or disturbances.

1.2.1. Additional Details on the Task Switching Paradigm

All stimuli were presented centrally in white colour on a black background. Each of the 396 trials started with the presentation of a 50 pt "Arial" font-sized cue 2 cm below a centrally presented fixation point. After 1300 ms of cue presentation, a central target stimulus (always a single digit between 1 and 9, except for 5) of either 50 pt or 80 pt "Arial" font size was presented together with the initial cue until a response was given, but no longer than for 2500 ms. The termination of cue and stimulus presentation was followed by 500 ms of fixation cross presentation, and then by a 1000 ms visual feedback provided in yellow font. In case of a correct response within 2500 ms after target onset, a 120 pt "+" was presented. In case of an incorrect response within 2500 ms after target onset, a 120 pt "-" was presented. In case of a missed response (i.e., either no response, or a response slower than 2500 ms) a 60 pt "Schneller!" (engl. "Faster!"), was presented. The inter-trial interval was a 300 ms presentation of the fixation cross. Every 33 trials, participants were offered to take a break.

In any given trial, one of three rules was in effect: The even/odd rule required a left button press if the target digit was odd, and a right button press if the target digit was even. The smaller/greater than 5 rule required a left button press if the target digit was smaller than 5, and a right button press if the target digit was greater than 5. The small/large font size rule required a left button press if the target digit was displayed at a font size 50 pt, and a right button press if the target digit was displayed at a font size of 80 pt. Of note, each rule was in effect equally for one third of the trials; left and right button presses were required equally often for each task rule. Additionally, all trials (except for the first trial after each pause) were rated as "repeat" or "switch" trials, depending on whether or not they repeated task rule from previous (n-1) trial.

The experiment consisted of two distinct blocks: During the cue-based block (the first 198 trials), the presented cues informed the participants which rule was in effect. The "NUM" cue (in German: "numerisch", translation: "numeric") indicated a smaller/greater than 5 rule. The "GER" cue (in German: "gerade", translation: "even") indicated an ven/odd rule. The "SG" cue (in German: "Schriftgröße", translation: "font size") indicated a small/large font size rule. The order of cues was randomized. In 50 percent of the rials, task rules had to be switched. During the memory-based block (the last 198 trials), participants had to memorize and update a fixed order of task rules in working memory starting with 3x NUM, then 3xGER, then 3xSG, then all over again, i.e. {NUM, NUM, NUM, GER, GER, GER, SG, SG, SG, NUM, NUM, NUM, GER, …}). In order to improve the comparability of cue-based and memory-based blocks, a dummy cue ("XXX") was displayed in all memory-based blocks. If participants lost track of the rules and/or responded incorrectly in three consecutive trials, regular cues (i.e., NUM, GER, or SG) were played instead of the "XXX" dummy cues for three subsequent trials. Due to the fixed sequence of task rules, two thirds of the trials were repeat trials and one third of the trials were switch trials.

To make sure that participants understood the task instructions correctly, there was an exercise of 18 cue-based trials and 18 memory-based trials prior to the experimental blocks. Participants took approx. 25 min to perform the task.

1.2.2. Additional Details on the Backward Inhibition Paradigm

All cue and target stimuli were centrally presented in white colour on a black background. Each trial started with the presentation of a geometrically shaped cue stimulus.

After 100 ms, a 50 pt "Arial" font-sized target stimulus (a single digit ranging from 1 to 9, excluding 5) was added in the center of the cue. There were three task rules: The odd/even rule ("task A"), which was indicated by a square cue, required a left key press if the target digit was odd and a right key press if the target digit was even. The smaller/larger than five rule ("task B"), which was indicated by a diamond cue, required a left key press if the target digit was smaller than five and a right key press if the target digit was larger than five. The double-press rule ("task D"), which was indicated by a triangle cue, required the participants to simultaneously press both response buttons. The combination of cue and target stimuli was presented until the participants responded with the required number of button presses. Participants were instructed to respond as fast and as accurately as possible and the 60 pt "Arial" font word "Schneller!" (engl. "Faster!") was shown above the triangle cue in case of double-press responses slower than 1000 ms. In case of too slow double-press responses in task D, the words "zu langsam!" (engl. "too slow!") were additionally presented in 60 pt Arial font from 30 ms to 550 ms after the response was given. In case of incorrect responses (i.e., wrong button press in tasks A and B, or non-simultaneous button presses that were more than 50 ms apart from each other, but faster than 1000 ms in task D), the word "FALSCH!" (engl. "WRONG!") was presented in 60 pt Arial font from 30 ms to 550 ms after the response was given. No visual feedback was given in case of correct responses. Responses were followed by a fixation cross which was presented for 1500 ms in the center of the screen. The response-stimulus interval (RSI) between the last given response and the onset of the cue n the following trial was set to 2500 ms.

The task consisted of 768 trials which were divided into 8 equally sized blocks and participants were offered to take a break after each block. To make sure that participants understood the task instructions correctly, there was an exercise of 24 trials prior to the experimental blocks. Participants took approx. 45 min to perform the task.

2. Results

2.1. Additional Details on the the Exclusion of Participants and Outlier Values

After collecting the data, we inspected the MRS data and excluded $n = 4$ participants due to lack of usable MRS data: $N = 3$ participants had to be excluded due to misplaced volumes of interest (VOIs) and/or due to technical errors in the MRS assessment. $N = 1$ participant was excluded because the visual inspection of all fitted spectra showed suboptimal fitting (a worse fit as compared to the spectra of all other included subjects). Out of the $n = 55$ remaining participants, there was incomplete MRS data in n = 5 participants for the following reasons: In $n = 2$ participants, MRS data for the ACC is missing because the MRS quantification failed to provide plausible values. In another $n = 3$ participants, we refrained from providing GABA+ levels for the ACC because the quantification war of poor quality (i.e., the error for GABA+ was > 15%). Finally, exploratory analyses revealed $n = 1$ extreme outlier in NAA-referenced GABA+ levels in the ACC, $n = 1$ extreme outlier in NAA-referenced GABA+ and Glx levels in the striatum, and $n = 1$ extreme outlier in GABA+/Glx ratios of the striatum and ACC. The respective values (but not the participants) were removed before running the statistical analyses detailed below.

As a second step, we also inspected the behavioral performance data collected in the two tasks. For the Task Switching Paradigm, $n = 2$ participants were excluded due to extreme (low) outliers in accuracy rates of less than 80% in at least one experimental condition. Yet, $N = 1$ of those two participants was also excluded due to poor MRS data quality, so that this only effectively resulted in the additional exclusion of just one participant. For the Backward Inhibition Paradigm, $n = 1$ participant was excluded because this person stopped participating before that task was started. $N = 2$ participants were excluded due to outlier (high) reaction times of more than 1100ms in at least one experimental condition. $N = 1$ participant was excluded due to outlier (low) accuracy of less than 35% in at least one experimental condition.

2.2. Additional Details on the Exclusion of Participants and Outlier Values (DKNTMN 0.45)

After collecting the data, we inspected the MRS data and made the same exclusions as for the analyses using the DKNTMN parameter of 0.15. The only differences were with respect to the exclusion of extreme outlier values: When using a DKNTMN parameter of 0.45, none of the participants' data had a poor quality of GABA quantification (i.e., the error for GABA+ was > 15%, which had been the case for n = 3 participants when using the DKNTMN parameter of 0.15). Also, there were no extreme outliers in NAA-referenced GABA+ levels in the ACC (previously $n = 1$), in NAA-referenced GABA+ and Glx levels in the striatum (previously $n = 1$), and in GABA+/Glx ratios of the ACC (previously $n = 1$) Still, an extreme outlier in GABA+/Glx ratios of the striatum for $n = 1$ participants was removed before running the statistical analyses detailed below.

2.2.1. Task Switching Paradigm: Behavioral Data with Sex as an Additional Factor

Upon reviewer request, we re-ran the repeated-measures ANOVAs for the behavioral data with sex as an additional factor. Doing so revealed the same pattern of significances and no significant effects of sex:

The ANOVA for accuracy revealed a significant main effect of condition ($F(1,52) = 4.903$, $p = 0.031$, $\eta^2_p = 0.086$), no significant main effect of block ($F(1,52) = 3.705$, $p = 0.060$, $\eta^2_p = .067$), and no significant interaction between block and condition ($F(1,52) = 1.692$, $p = 0.199$, $\eta^2_p = 0.032$). Neither the main effect of sex, nor any of its interaction effects were significant (all $F \leq 1.776$; all $p \geq 0.188$).

The ANOVA for RTs revealed a significant main effect of block ($F(1,52) = 6.092$, $p = 0.017$, $\eta^2_p = 0.105$), a significant main effect of condition ($F(1,52) = 73.970$, $p < 0.001$, $\eta^2_p = 0.587$), and a significant interaction between block and condition ($F(1,52) = 23.734$, $p < 0.001$, $\eta^2_p = 0.313$). Neither the main effect of sex, nor any of its interaction effects were significant (all $F \leq 1.180$; all $p \geq 0.282$).

Since we found no sex differences in behaviour, we refrained from including this factor in the main manuscript and further refrained from re-analyzing the MRS data with sex as an additional factor.

2.2.2. Task Switching Paradigm: Additional MRS Measures (DKNTMN 0.45)

As found using the DKNTMN parameter of 0.15, the ACC absolute tCr significantly correlated with the RT switching effect in the memory condition (i.e., memory/switch mi nus memory/repetition; $r = .314$, $p = .023$) and add- on Bayesian analyses provided anecdotal evidence in favor of the alternative hypothesis ($BF_{01} = 0.711$). Additionally (and other than for a DKNTMN of 0.15, we also found significant correlations between the ACC absolute tCr and the RT in repeat trials of the cue condition ($r = -0.286$; $p = .040$; $BF_{01} = 1.131$) and between the striatal absolute tCr and the accuracy in switch trials of the cue condition ($r = -0.341$; $p = .012$; $BF_{01} = 0.395$). All other correlations between absolute tCr and behavioral values were non-significant (all $p \geq .089$; all $BF_{01} \geq 2.104$). In contrast to this and as found using a DKNTMN parameter of 0.15, NAA reference values did not significantly correlate with any behavioral measure in the task switching paradigm (all $p \geq .059$) and all Bayesian analyses for the NAA correlations were more in favor of the null hypothesis than of the alternative hypothesis (all $BF_{01} \geq 1.574$). Based on this, we decided to again reference Glx and GABA+ to NAA for the following analyses.

To investigate whether MRS-assessed transmitter levels correlated with performance in the task switching paradigm, we ran linear correlation analyses. As for the DKNTMN parameter of 0.15, this also revealed a single significant correlation, but this time it was found between Glx in the ACC and the task switching effect (i.e., switch minus repeat) in hit RTs of the memory block ($r = 0.308$; $p = 0.028$). Yet again, an add-on Bayesian analysis only provided anecdotal evidence for the alternative hypothesis ($BF_{01} = 0.790$). Aside from this effect, there were no other significant correlations between any of the assessed behav ioral parameters and GABA+/NAA or Glx/NAA or GABA+/Glx in either the ACC or striatum (all $p \geq 0.057$). Of note, the add- on Bayesian analyses for all of the other correlations

were also more in favor of the null hypothesis than the alternative hypothesis, even though evidence was sometimes only on an anecdotal level (all $BF_{01} \geq 1.528$).

Again, we further performed multiple linear regression analyses with all MRS values as independent and each single behavioral measure as separate dependent variable to further confirm our results of the correlation analyses. As for the analyses using the DKNTMN parameter of 0.15, we neither found the GABA+/NAA MRS measures (all $p \geq 0.214$, adj. $R^2 \leq 0.023$), nor the Glx/NAA MRS measures (all $p \geq 0.141$, adj. $R^2 \leq 0.039$), nor the GABA+/Glx ratio measures (all $p \geq 0.169$, adj. $R^2 \leq 0.033$) to be significant predictors for any behavioral measure. Furthermore, additional Bayesian regression analyses indicated that there is at least strong evidence for the null hypothesis compared to the alternative hypothesis for the GABA+/NAA MRS measures (all $BF_{01} \geq 12.001$), at least substantial evidence for the Glx/NAA MRS measures (all $BF_{01} \geq 7.935$) and the GABA+/Glx ratio measures (all $BF_{01} \geq 9.296$).

2.2.3. Backward Inhibition Paradigm: Behavioral Data with Sex as an Additional Factor

Upon reviewer request, we re-ran the repeated-measures ANOVAs for the behavioral data with sex as an additional factor. Doing so revealed the same pattern of significances and no significant effects of sex:

The ANOVA for accuracy revealed no significant effect of condition ($F(1,49) = 2.346$, $p = 0.132$, $\eta^2_p = 0.046$), no significant effect of sex ($F(1,49) = 0.016$, $p = 0.900$, $\eta^2_p < 0.001$), and no significant interaction between condition and sex ($F(1,49) = 3.209$, $p = 0.079$, $\eta^2_p < 0.061$).

The ANOVA for RTs revealed a significant main effect of condition ($F(1,49) = 43.689$, $p < 0.001$, $\eta^2_p = 0.471$), but no significant effect of sex ($F(1,49) = 0.106$, $p = 0.746$, $\eta^2_p = 0.002$), and no significant interaction between condition and sex ($F(1,49) = 0.566$, $p = 0.455$, $\eta^2_p < 0.011$).

Since we found no sex differences in behavior, we refrained from including this factor in the main manuscript and further refrained from re-analyzing the MRS data withsex as an additional factor.

2.2.4. Backward Inhibition Paradigm: Additional MRS Measures (DKNTMN 0.45)

As found using the DKNTMN parameter of 0.15, correlating absolute tCr and NAA concentrations with behavioral measures revealed only non-significant results (all $p \geq 0.140$) and all Bayesian analyses were more in favor of the null hypothesis than of the alter native hypothesis (all $BF_{01} \geq 3.098$), thus suggesting that there was no meaningful correlation between either tCR or NAA and performance. Against this background, we again decided to use NAA-referenced values for further analyses in the BI paradigm as well, as this yields better comparability with the task switching paradigm.

We also correlated GABA+/NAA, Glx/NAA, and the GABA+/Glx ratio with behavioral RT measures. As for the analyses using a DKNTMN parameter of 0.15, found no significant correlations between any of the assessed behavioral parameters and GABA+/NAA or Glx/NAA or GABA+/Glx in either the ACC or striatum (all $p \geq 0.112$) and add-on Bayesian analyses provided evidence for the null hypothesis (all $BF_{01} \geq 2.721$), even though it was sometimes only anecdotal.

Again, we further performed multiple linear regression analyses with the MRS values as independent and each single behavioral measure as separate dependent variables to further confirm our results of the correlation analyses. As for the analyses with the DKNTMN parameter ot 0.15, we neither found the GABA+/NAA MRS measures (all $p \geq 0.253$, adj. $R^2 \leq 0.017$), nor the Glx/NAA MRS measures (all $p \geq 0.261$, adj. $R^2 \leq -0.016$), nor the GABA+/Glx ratio measures (all $p \geq 0.621$, adj. $R^2 \leq -0.023$) to be significant predictors of any behavioral measure. Further Bayesian regression analyses were also more in favour of the null hypothesis than of the alternative hypothesis and indicated that there was at least strong evidence for the null hypothesis for the GABA+/NAA MRS measures (all BF_{01}

≥13.361) and the Glx/NAA MRS measures (all $BF_{01} \geq 13.762$), and at least very strong evidence for the null hypothesis for the GABA+/Glx ratio measures (all $BF_{01} \geq 31.812$).

2.3. Summary of Additional Results (DKNTMN 0.45)

As for the MRS data obtained using a DKNTMN parameter of 0.15, our add- on analyses using a DKNTMN of 0.45 did not confirm our hypotheses that GABA levels, glutamate levels, or their ratio in the ACC or striatum correlate with task switching performance, or with BI: Correlation analyses did not reveal significant correlations of either transmitter, or their ratio, with any of the relevant behavioral measures, except for a single correlation between Glx in the ACC and the response time task switching effect (i.e., switch minus repeat) in the memory block. Yet, it needs to be noticed that this result was obtained without correcting for multiple testing and Bayesian analyses failed to provide convincing evidence for the alternative hypothesis being true. As GABA and glutamate levels as well as their ratio did also not predict any of the behavioural measures in the subsequent multiple linear regression analyses, we deem it safe to state that we found no functionally relevant effects.

References

78. Marjańska, M.; Lehéricy, S.; Valabrègue, R.; Popa, T.; Worbe, Y.; Russo, M.; Auerbach, E.J.; Grabli, D.; Bonnet, C.; Gallea, C.; et al. Brain Dynamic Neurochemical Changes in Dystonic Patients: A Magnetic Resonance Spectroscopy Study. *Mov. Disord.* **2013**, *28*, 201–209. https://doi.org/10.1002/mds.25279.
79. Tremblay, S.; Beaulé, V.; Proulx, S.; Lafleur, L.-P.; Doyon, J.; Marjańska, M.; Théoret, H The Use of Magnetic Resonance Spectroscopy as a Tool for the Measurement of Bi-Hemispheric Transcranial Electric Stimulation Effects on Primary Motor Cortex Metabolism. *J. Vis. Exp.* **2014**. https://doi.org/10.3791/51631.
80. Provencher, S.W. Estimation of Metabolite Concentrations from Localized in Vivo Proton NMR Spectra. *Magn. Reson. Med.* **1993**, *30*, 672–679.
81. Kreis, R.; Bolliger, C.S. The Need for Updates of Spin System Parameters, Illustrated for the Case of γ-Aminobutyric Acid. *NMR Biomed.* **2012**, *25*, 1401–1403. https://doi.org/10.1002/nbm.2810.
